# Periodontal Regeneration Using Strontium-Loaded Mesoporous Bioactive Glass Scaffolds in Osteoporotic Rats

**DOI:** 10.1371/journal.pone.0104527

**Published:** 2014-08-12

**Authors:** Yufeng Zhang, Lingfei Wei, Chengtie Wu, Richard J. Miron

**Affiliations:** 1 The State Key Laboratory Breeding Base of Basic Science of Stomatology (Hubei-MOST) & Key Laboratory of Oral Biomedicine Ministry of Education, Wuhan University, Wuhan, People's Republic of China; 2 State Key Laboratory of High Performance Ceramics and Superfine Microstructure, Shanghai Institute of Ceramics, Shanghai, People's Republic of China; 3 Faculté de medecine dentaire, Université Laval, Québec, Canada; University of Toronto, Canada

## Abstract

Recent studies demonstrate that the rate of periodontal breakdown significantly increased in patients compromised from both periodontal disease and osteoporosis. One pharmacological agent used for their treatment is strontium renalate due to its simultaneous ability to increase bone formation and halt bone resorption. The aim of the present study was to achieve periodontal regeneration of strontium-incorporated mesoporous bioactive glass (Sr-MBG) scaffolds in an osteoporotic animal model carried out by bilateral ovariectomy (OVX). 15 female Wistar rats were randomly assigned to three groups: control unfilled periodontal defects, 2) MBG alone and 3) Sr-MBG scaffolds. 10 weeks after OVX, bilateral fenestration defects were created at the buccal aspect of the first mandibular molar and assessed by micro-CT and histomorphometric analysis after 28 days. Periodontal fenestration defects treated with Sr-MBG scaffolds showed greater new bone formation (46.67%) when compared to MBG scaffolds (39.33%) and control unfilled samples (17.50%). The number of TRAP-positive osteoclasts was also significantly reduced in defects receiving Sr-MBG scaffolds. The results from the present study suggest that Sr-MBG scaffolds may provide greater periondontal regeneration. Clinical studies are required to fully characterize the possible beneficial effect of Sr-releasing scaffolds for patients suffering from a combination of both periodontal disease and osteoporosis.

## Introduction

Osteoporosis is a worldwide chronic disease which now affects over 200 million people worldwide characterized by low bone mass, poor bone strength and microarchitectural deterioration of bone [1]. The primary cause is governed by the imbalance between bone forming osteoblasts and bone resorbing osteoclasts commonly resulting from postmenopausal oestrogen deficiency [2], [3]. At present, the two major therapies include the use of anabolic agents such as parathyroid hormone that stimulate bone formation, and anti-resorptive agents including bisphosphonates, calcitonin, raloxifene, RANKL inhibitors and estrogen which act by inhibiting osteoclast differentiation and activity [4], [5]. In relation to periodontal tissues, osteoporosis is believed to contribute to periodontal breakdown given that it may increase bone resorption and prevent proper healing ultimately increasing the severity of the pre-existing periodontal disease [6]–[8]. Diminished bone density as seen in osteoporotic bone leads to an increase in susceptibility towards alveolar bone loss and further complicates regenerative periodontal procedures.

One agent that is clinically used to prevent bone loss in osteoporotic patients is strontium renalate [9]–[14]. Studies have demonstrated that it simultaneous acts by both increasing bone formation and decreasing bone resorption [15]–[20] thus demonstrating increases in bone mineral density in the lumbar spine, the femoral neck and in total hip reconstruction following its use in clinical trials [21]–[25]. Its dual mechanism of action makes it advantageous over other leading therapies. While the great majority of research in the field of osteoporosis is currently focused on the preventative measures of disease progression, less study on the therapeutic effect of local transplantation of bioactive scaffolds has been investigated following osteoporotic-related fractures. The advancements in tissue engineering over the last several decades warrant the discovery and application of new therapeutic options carrying bioactive and pharmacological agents within scaffolds capable of guiding cell tissue response upon implantation.

One bone grafting material that has gained awareness in recent years is mesoporous bioactive glass (MBG); a synthetic bone graft capable of bone regeneration [26], [27]. Recently it has been demonstrated that the chemical composition in MBG (CaO-P2O5-SiO2) improves the in vitro cell activity of cells seeded on MBG scaffolds and improves bone osseointegration in vivo [28], [29]. Furthermore, MBG scaffolds have optimal degradation properties making them slowly resorbed over time and replaced by native bone and provide the additional benefit of easily carrying pharmacological agents capable of being released over time to the surrounding tissues [28]–[31]. Recently we have demonstrated that the advantages of MBG scaffolds incorporated with trace element Strontium (Sr-MBG) were a suitable scaffold for the delivery of strontium to bone defects in a rat osteoporosis model [30], [31]. The aim of the present study was to determine if the advantages of Sr-containing mesoporous bioactive glass scaffolds could also be advantageous for the repair of alveolar bone defects created in periodontal tissues. Acute type fenestration defects were created on the buccal aspect of first mandibular molars in 15 ovariectomised rats to generate an osteoporotic phenotype. Healing was assessed 4 weeks post implantation by micro-CT, hematoxylin and eosin staining, and Mason staining.

## Materials and Methods

### Animals and surgical procedures

15 mature female Wistar rats (10 weeks old, mean body weight 230 g) were purchased and used for this study with all handling and surgical procedure approved by the Ethics Committee for Animal Research, Wuhan University, China. Animals had food and water ad libitum with constant temperature at 22 degrees Celsius.

After one week for acclimatizing to the new laboratory surroundings, an osteoporosis animal model was carried out by bilateral ovariectomy (OVX) under sterile conditions with a minimally invasive surgical technique as previously described [32], [33]. Briefly, when general anesthesia by intraperitoneal injection of chloral hydrate (10%, 4 ml/kg body weight) was achieved, rats were operated with 10 mm linear bilateral lumbar lateral skin incisions. Then the enterocoelia was exposed by blunt dissection of muscle and peritoneum. The bilateral ovaries were removed gently following ligation of the ovarian artery and vein. Then the overlying muscles and epithelial tissues were sutured in multi-layers. Postoperatively, penicillin (40,000 IU/ml, 1 ml/kg) was injected for 3 days and there was no sign of inflammation or other notable anomaly.

Periodontal fenestration defects (standardized with 2.8 mm in length, 1.4 mm in height and ≈0.5 mm in deep) were created 2 months later when an osteoporosis model was established as previously described [34]. Briefly, under general anesthesia, rats were subjected to bilateral extra-oral incision at the base of the mandible. The buccal mandibular bone overlying the first molar roots was removed to create a defect (2.8 mm in length, 1.4 mm in height and ≈0.5 mm in depth) using a size-4 round bur. The procedure was performed under an operating microscope to avoid perforation of intraoral mucosa. The roots of the first molar were carefully denuded of their periodontal ligament, overlying cementum, and superficial dentin. The height was standardized to the width of the round bur (diameter 1.4 mm) and extended longitudinally to either side. Then, bilateral defects were created in 15 animals for a total of 30 defects and divided into three groups of 10 defects as follows: 1) non-treated control, 2) MBG alone and 3) Sr-MBG group. Porous strontium-incorporated mesopore-bioglass (Sr-MBG) scaffolds were prepared according to the method as previously described [35]. Following implantation of scaffolds, the muscle and the skin were repositioned and sutured separately. Postoperatively, penicillin (40,000 IU/ml, 1 ml/kg) was injected intramuscularly for 3 days. Four weeks after surgery, the animals were sacrificed by an overdose of chloral hydrate and samples were removed and prepared for analysis.

### µCT analysis

The samples were fixed in 4% formaldehyde for 12 h at 4°C. A µCT imaging system (µCT50, Scanco Medical, Bassersdorf, Switzerland) was used to reveal new bone formation within the defect region. Scanning parameters was performed at 70 kV and 114 µA with a thickness of 0.048 mm per slice in medium-resolution mode. For 3D reconstruction, the mineralized bone tissue was differentially segmented with a fixed low threshold (value  = 212). Representative sections were cut out from buccal and mesial-distal view. After 3D reconstruction, the bone volume faction (BV/TV) was determined in defect regions to evaluate new bone formation, using a protocol provided by the manufacturer.

### Histological analysis

The mandibles were decalcified in 10% EDTA for 4 weeks. Gradient dehydration was performed for embedding in paraffin followed by perpendicular sectioning to the long axis of the molar roots as previously described [36]. Serial sections of 5 µm were cut and mounted on polylysine-coated slides and then performed for H&E staining, Masson trichrome staining (Sigma #HT15; Sigma-Aldrich, St. Louis, USA.) and tartrate-resistant acid phosphatase (TRAP) staining (Sigma #387A; Sigma-Aldrich, St. Louis, USA.) in accordance with the manufacturer's protocol. For histomorphometry, six individual sections were selected from three different locations, which were situated in the middle, coronal and apical levels of the defect (with 400 µm apart from the central). The histometric measurements were determined by processing the images, which were captured with an Olympus DP72 microscope, in Adobe Photoshop CS5 (Adobe Systems, Inc.). New mineralized tissue was identified by Masson trichrome staining and the defect fill was defined as the ratio of area of new mineralized tissue within bony envelope and the total defect area. The number of TRAP-positive cells was performed in a 500 µm square of the defect area.

### Statistical analysis

All data analysis was performed using SPSS software and statistically significant values were adopted as p <0.05. Based on the sample size, a Kolmogorov-Smirnow Test was used to confirm the asymptotic normality of our data. For the bone density, bone thickness and percentage of angiogenesis, mean and standard deviation (SD) were calculated and statistical inference was made by one-way ANOVA and Student t-test (Newman Keuls Test).

## Results

### Establishment of rat osteoporotic model

**Figure 1:**
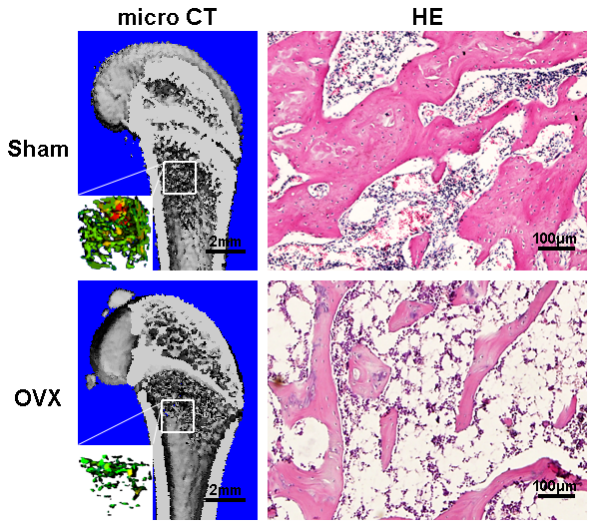
Establishment of rat osteoporotic model created by OVX. 3D µ-CT images of normal bone and osteoporotic bone as well as their representative H&E staining.

Both 2D representation and 3D µ-CT images of the ovariectomized rat animal model was confirmed by demonstrating a decrease in trabecular bone volume, thickness and density (Fig. 1). Furthermore, OVX animals also demonstrated a significant increase in trabecular separation, a reduced thickness of cortical bone and enlarged marrow cavities when compared to control animals (Fig. 1). After analysis of 3D reconstruction, BV/TV in the distal femur region was significantly decreased in OVX rats confirming the established osteoporotic model (data not shown). H&E staining also supported the establishment of an osteoporotic model by demonstrating evident deterioration of trabecular patterns with fat-rich bone marrow-like tissue in ovariectomized rats (Fig. 1).

### 3D reconstruction observations and analysis

**Figure 2:**
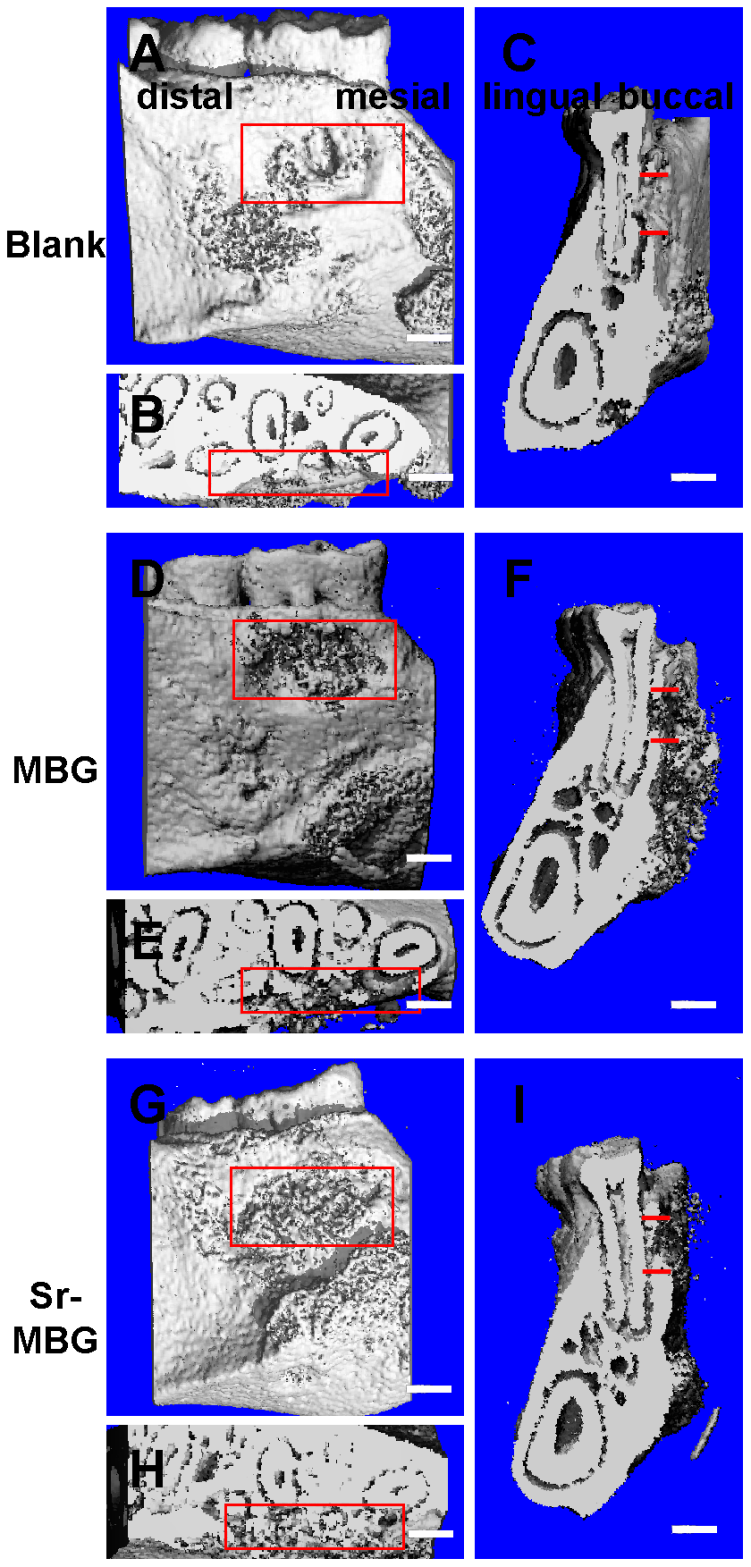
The overall state of bone regeneration was exhibited by 3D reconstruction. The red box represented the original extent of surgical defect in general. A, D and G: buccal view; B, E and H: screenshot in horizontal; C, F and I: screenshot in vertical. Bar: 1 mm.

**Figure 3:**
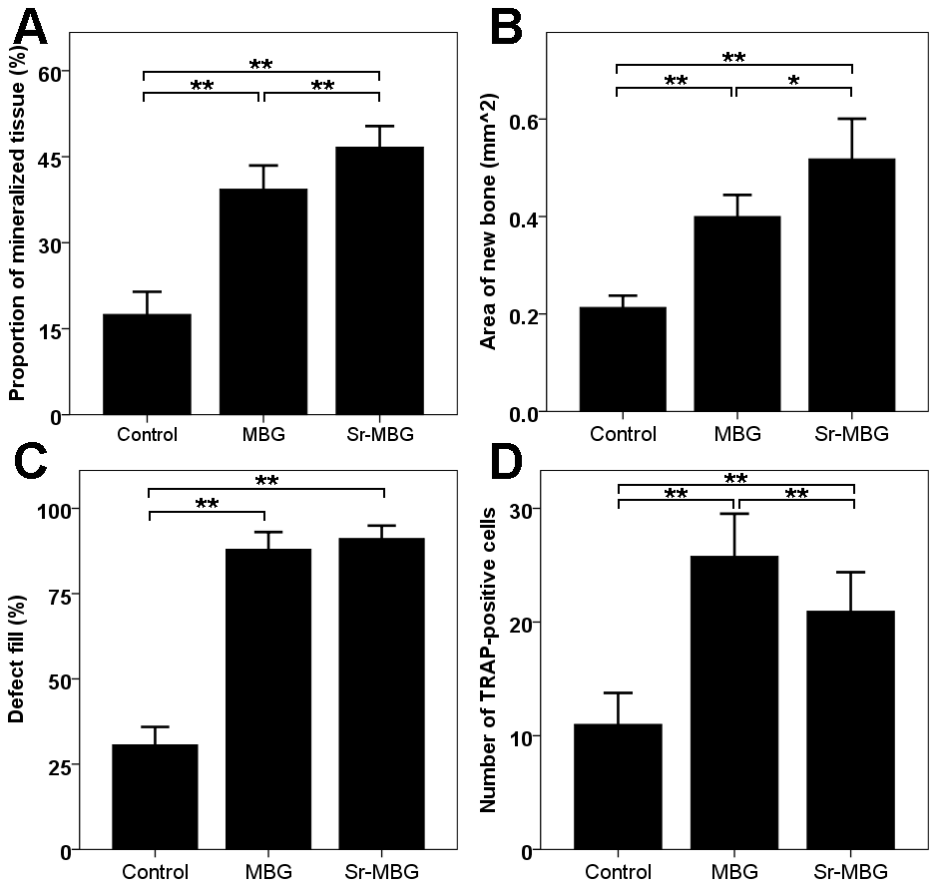
Statistical analysis of periodontal regeneration: A) proportion of mineralized tissue from horizontal cutaway view of 3D construction, B) area of new bone from H&E stain, C) defect fill from Masson trichrome stain and D) the number of TRAP-positive cells from TRAP stain. Results were given by mean ±SD. *: p<0.05, **: p<0.01.

Following establishment of an osteoporotic model, representative images of osteogenesis in the periodontal fenestration defect performed by 3D reconstruction for each group were shown in ways of buccal holistic view (Fig. 2 A, D and G) and cutaway view from both horizontal (Fig. 2 B, E and H) and vertical (Fig. 2 C, F and I) directions. Only minimal regenerated bone was visible in the control group, where both defects treated with scaffolds demonstrated newly formed bone following implantation with either MBG or Sr-MBG. The groups receiving Sr-MBG scaffolds exhibited statistically higher newly mineralized tissue when compared to the other modalities (Fig. 3A; 17.50±3.94%, 39.33±4.13% and 46.67±3.67%, P<0.01, for control, MBG and Sr-MBG, respectively).

### Histological observation and analysis

**Figure 4:**
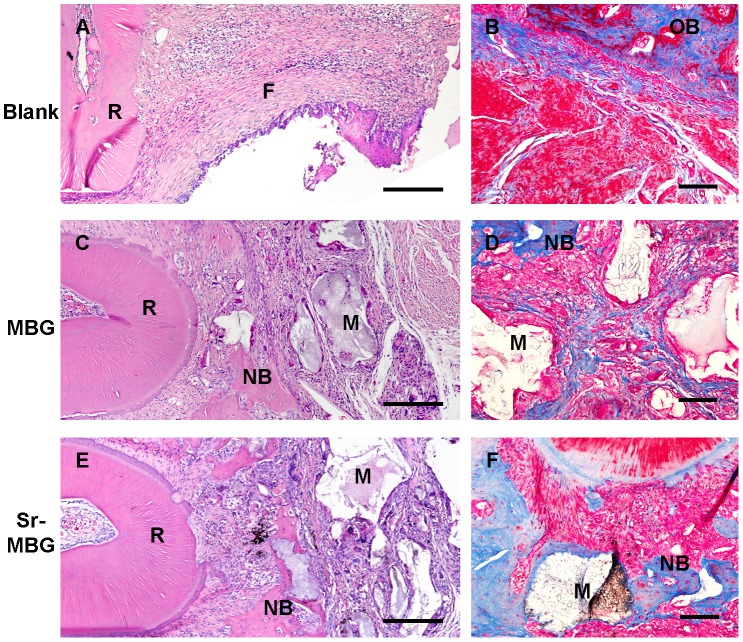
Representative sections of H&E stain (A, C and E) revealed the osteogenesis among the defects. New bone was visible in the area adjacent to the old bone and implanted scaffolds. Representative sections of Masson trichrome stain (B, D and F) revealed the compound of collagen in the osteogenic active zone. Bar: 200 µm. R: Root, F =  Fibroblasts, NB =  New bone, OB =  Old bone, M =  Material.

The histological observation demonstrated that little bone was observed in control defects (Fig. 4A). Within the defects receiving MBG or Sr-MBG scaffolds, a fibrous tissue of woven bone occurred around at the edges as well as around the scaffolds themselves (Fig. 4C and E). Consistently, no new cementum was observed on the root surface among all three groups at a time point of 28 days. It was noted that osteoblasts clustered on the surface of MBG and Sr-MBG and participated aided in the osteogenesis and mineralization (Fig. 4D and F). Noteworthy, more mature alveolar bone was generated around the Sr-MBG when compared to MBG scaffolds. Histomorphometric analysis additionally revealed that the area of new bone was statistically higher in Sr-MBG group (0.52±0.08 mm^2^) when compared to other modalities (Fig. 3B). Interestingly, little difference was observed between MBG and Sr-MBG for defect fill indicating that the speed of new bone formation was slightly faster in the defects receiving Sr-MBG scaffolds (Fig. 3C).

**Figure 5:**
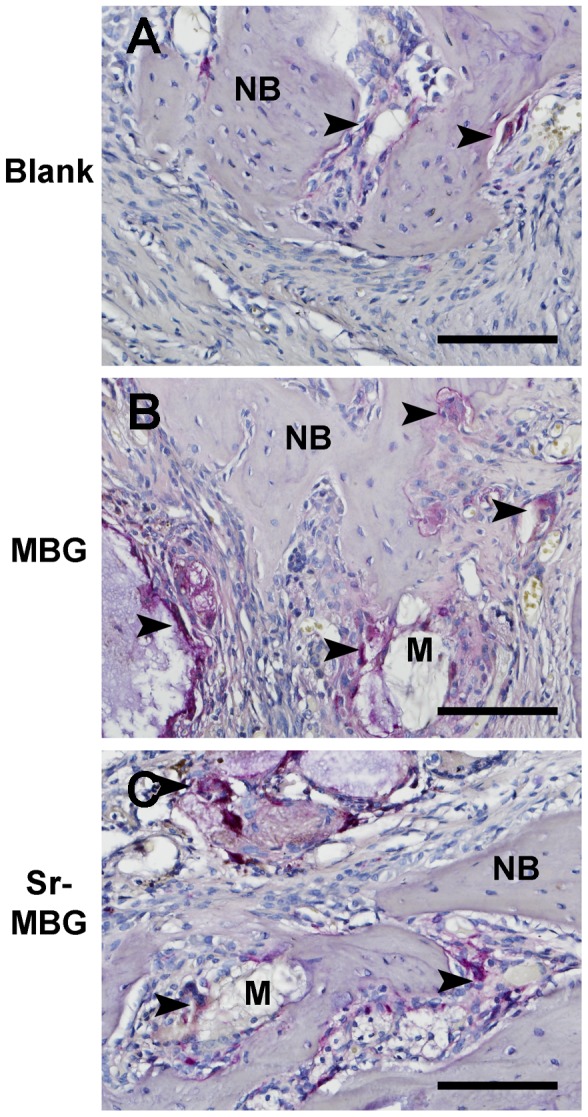
TRAP-staining for A) control, B) MBG alone and C) Sr-MBG scaffolds. Positive cells were visible in the surface of bone (control, MBG and Sr-MBG) and scaffolds (MBG and Sr-MBG). Bar: 100 µm. Arrows depict areas of TRAP-staining, NB =  New bone, M =  Material.

TRAP-staining was used to determine the number of multi-nucleated cells laying at the edges of the alveolar bone defect in all three groups (Fig. 5). Large multi-nucleated cells stained for TRAP were observed around both MBG and Sr-MBG scaffolds. The addition of Sr to MBG significantly decreased the number of TRAP-positive stained cells when compared to MBG scaffolds alone. Both scaffold groups showed higher values when compared to control unfilled defects (Fig. 3D, P<0.01).

## Discussion

The aim of the present study was to investigate the effect of Sr-containing scaffolds on the biological response of periodontal defects created in OVX rats. As the prevalence of osteoporosis continues to rise along with a high number of patients with periodontal disease in the elderly population, the need for treatment modalities directed at patients suffering from precise combination of diseases remains prominent. The ability to direct therapy to meet the patient's individual needs remains the clinician's optimal and desired outcome. For these reasons, the goal of the present study was to utilize the therapeutic benefits from the active pharmacological agent in strontium renalate, and fabricate a scaffold containing Sr to assist in the regeneration of periodontal defects in osteoporotic rats. Recently we have demonstrated that the release kinetics and in vitro behavior of these scaffolds demonstrates ideal release of Sr ions over time and supports osteoblast proliferation and differentiation as well as bone regeneration in a rat femur defect model [31]. The goal therefore of the present study was to further investigate the ability for these scaffolds to fully regenerate the more complicated periodontal defect in OVX animals.

The benefit and future incorporation of Sr into pharmacological agents and biomaterials stems from previous studies indicating that Sr ions is a safe and effective way to stimulate proliferation and differentiation of bone mesenchymal stem cells, osteoblasts and periodontal ligament cells harvested [19], [37]. It has previously been demonstrated that Sr significantly influences osteoblastic differentiation by increasing alkaline phosphatase activity, real-time PCR for ALP and osteocalcin mRNA expression, and alizarin red staining for mineralization. Furthermore, recent investigations have also demonstrated a positive correlation of Sr incorporation into biomaterials [38], [39].

In order to verify our hypothesis that Sr would be advantage in combination with a carrier scaffold, we created Sr-MBG scaffolds and implanted them in acute type periodontal defects in ovariectomised rats to mimic the osteoporotic phenotype. The micro-CT analysis demonstrated that Sr-MBG scaffolds induced more mineralization than MBG scaffolds alone or control samples. The morphological and histormorphometry analysis based on the HE and Mason staining suggested that the Sr-MBG stimulated a more effectively osteoconductive and anti-osteoporotic phenotype which increased the speed and quality of bone regeneration. Furthermore it was shown from TRAP staining that the number of multi-nucleated giant cells was significantly reduced when Sr was administered in MBG scaffolds (Fig. 4). This finding is in accordance with studies from the literature that demonstrate that Sr is able to suppress osteoclastogenesis [40]–[44]. It has been shown that Sr is able to reduce osteoclast resorption in vitro [40] by decreasing receptor activator of nuclear factor-KappaB ligand (RANKL)-induced osteoclast differentiation [41], [44].

The incorporation of strontium into biomaterials has become a hot topic in recent years. Investigators have now demonstrated positive results for the incorporation of Sr into calcium phosphate [45], [46], osseointegration of titanium implants [47], [48], hydroxyappatite implants [49]–[51] and bioactive glass [52]. These investigations utilize the bone formation capabilities of Sr not only for osteoporotic related defects and patients but also for the general public. Thus, it is plausible that the use of Sr may become mainstream within the next decade for a large variety of dental application. The desirable outcome of simultaneously improving bone formation while decreasing bone resorption by incorporating Sr into biomaterials presents many future options for a large variety of applications.

In conclusion, the results from the present study demonstrates that Sr-releasing MBG scaffolds are capable of significantly increasing alveolar bone regeneration in periodontal tissues in OVX rats 28 days post-surgery when compared to control and MBG groups. To the best of our knowledge, we demonstrate for the first time the ability for Sr-containing scaffolds to support bone formation in periodontal tissues in vivo. The scaffolds utilized in this study demonstrate that the release of Sr2+ ions is capable of improving osteoblast function by increasing new bone formation. Simultaneously, the release of Sr also significantly decreased the number of multi-nucleated osteoclasts as demonstrated by TRAP staining. Taken together, these results suggest that the use of Sr-containing scaffolds may provide greater defect healing in osteoporotic related periodontal defects. Additional clinical studies are required to fully characterize the possible beneficial effect of Sr-MBG scaffolds for patients suffering from both periodontal disease and osteoporosis.
